# P08-07 The effects of an integrated approach to a worksite intervention on the mental health and wellbeing of cleaners: A randomized stepped wedge worksite study

**DOI:** 10.1093/eurpub/ckac095.120

**Published:** 2022-08-29

**Authors:** Vivian Rueskov Poulsen, Ole Steen Mortensen, Margrethe Bordado Sköld, Sanna Koch Autrup, Brian Oldenburg, Korshøj Mette

**Affiliations:** 1 Department of Occupational and Social Medicine, Holbæk Hospital, Holbæk, Denmark; 2 Section of Social Medicine, Department of Public Health, University of Copenhagen, Copenhagen, Denmark; 3 Baker Heart and Diabetes Institute, La Trobe University, Melbourne, Australia

**Keywords:** integrated approach, worksite intervention, mental health, cleaners, stepped wedge

## Abstract

**Background:**

Despite an intensive focus on worker health over the last three decades, the prevalence of work-related diseases remain largely unchanged in Denmark and internationally. In recent years, American and Australian researchers have developed new approaches to integrate health promotion, prevention of work-related disease and organization of work. The aim of this study was to examine whether an Integrated Approach to Health, Wellbeing and Productivity at Work (ITASPA) intervention would promote the mental health and wellbeing of workers.

**Methods:**

Two worksites were recruited and offered the intervention and 76 cleaners agreed to participate in the scientific evaluation. At each worksite, employees developed initiatives to improve the psychosocial work environment, building on top of existing work environment programs, practices and procedures. The developed initiatives did not hold any physical activity components. The intervention was planned to run for 12 months, and this analysis presents data from the first of four follow-ups. Data on mental health and wellbeing were obtained using the SF12 and Orebro questionnaires. Using a stepped wedge design, participants functioned as their own controls. Data were analysed using a linear mixed model with random slope and intercept. Intercorrelation of repeated measurements was included in the models as random effect.

**Results:**

The results showed significant decrease in sleeping problems (-4.04, 95% CI, -5,32- -2.75) after the intervention. Moreover, there was a non-significant increase in the amount of time participants had felt relaxed and calm (0.33, 95% CI, -0.10-0.96) and a small non-significant decrease in the amount of time participants had felt sad (-0.004, 95% CI, -0.42-0.41). Finally, results showed a small non-significant increase in how much physical pain had challenged the daily work (0.04, 95% CI, -0.21-0.30).

**Conclusion:**

The findings show that ITASPA intervention led to significantly reduced sleep problems. There was a tendency of improved self-ratings of feeling relaxed and calm as well as reduced feeling sadness. The intervention did not decrease ratings of whether physical pain challenged the daily work, however, the worksites decided to focus on the psychosocial work environment and thus changes in mental health are expected to show greatest effects.

